# Beyond the Heart: The Neuroprotective Potential of Nebivolol in Acute Myocardial Infarction

**DOI:** 10.3390/life15121880

**Published:** 2025-12-09

**Authors:** Guldem Mercanoglu, Ozge E. Bamac, Gulbin Sennazlı, Rivaze Kalaycı, Fehmi Mercanoglu

**Affiliations:** 1 Department of Pharmacology, Hamidiye Pharmacy Faculty, University of Health Sciences, Istanbul 34668, Türkiye; 2 Department of Pathology, Veterinary Faculty, Istanbul University-Cerrahpasa, Istanbul 34320, Türkiye; oerdogan@iuc.edu.tr (O.E.B.); ayyildiz@istanbul.edu.tr (G.S.); 3 Department of Laboratory Animal Science, Aziz Sancar Institute of Experimental Medicine, Istanbul University, Istanbul 34104, Türkiye; rivaze@istanbul.edu.tr; 4 Department of Cardiology, Istanbul Medical Faculty, Istanbul University, Istanbul 34104, Türkiye; fmercan@istanbul.edu.tr

**Keywords:** myocardial infarction, nebivolol, neuroprotection, neuroinflammation, nitric oxide, heart-brain axis, beta-blocker

## Abstract

Myocardial infarction (MI) triggers complex heart–brain interactions that increase the risk of stroke, cognitive decline, and mortality. Neuroinflammation and oxidative stress serve as critical mediators of these complications. We evaluated the neuroprotective effects of nebivolol, a β-blocker with nitric oxide-releasing properties, during acute MI. Male Sprague-Dawley rats were divided into sham-operated controls, MI-induced controls, and MI groups treated with oral nebivolol or intravenous loading followed by oral nebivolol. MI was induced by left anterior descending coronary artery ligation. Cardiac function was assessed by echocardiography and hemodynamic measurements. Brain tissues were analyzed for proinflammatory cytokines, oxidative stress markers, and histopathological changes. Nitric oxide synthase (NOS) isoform expression was evaluated by immunohistochemistry. MI induced significant neuroinflammation in the cerebral cortex and hippocampus, characterized by elevated cytokines, increased oxidative stress, upregulated iNOS expression, and altered histological patterns (necrosis, astrocytosis, gliosis, demyelination). Intravenous nebivolol significantly reduced these neuroinflammatory markers, normalized cytokine levels, prevented structural brain changes, and attenuated iNOS expression, while oral administration showed minimal effects. Both routes preserved cardiac function without affecting infarct size. These findings demonstrate that nebivolol, particularly via intravenous administration, provides significant NO-dependent neuroprotection during acute MI, supporting its potential as a dual-action therapeutic strategy targeting both cardiac and neurological complications.

## 1. Introduction

Myocardial infarction (MI) is more than an isolated cardiac event. It triggers a complex set of pathophysiological changes that affect both the cardiovascular and cerebrovascular systems. This intricate heart–brain interaction has profound clinical implications, as MI patients face substantially elevated risks of stroke, depression, and mortality that persist long after initial hospital admission [1,2,3,4]. Understanding and targeting these interconnected pathways is critical in cardiovascular medicine.

The acute phase of MI initiates immediate and far-reaching neurological consequences through multiple mechanisms [5,6]. These acute changes lead to chronic neurological consequences, such as progressive gray matter atrophy [7], cognitive decline with increased dementia risk [8], and widespread white matter abnormalities [9].

The molecular mechanisms underlying these heart–brain interactions involve multiple interconnected pathways. Among these mechanisms, inflammation serves as a central mediator, exerting a dual role that influences both left ventricular remodeling and brain pathology following MI [10,11,12]. This inflammatory nexus represents an attractive therapeutic target, as interventions that modulate inflammatory responses could provide dual cardioprotective and neuroprotective benefits.

Our previous research demonstrated that nebivolol, a third-generation selective β-blocker with unique nitric oxide (NO)-releasing properties, exhibits cardioprotective effects in preventing left ventricular remodeling after MI [13,14,15]. Nebivolol exhibits a dual mechanism of action that integrates β1-adrenergic blockade with the activation of endothelial NO synthase. This unique pharmacological profile makes it an exemplary candidate for effectively addressing both cardiac and neurological complications associated with MI. The NO-mediated effects of nebivolol may be particularly relevant to neuroprotection, given NO’s crucial roles in maintaining cerebral blood flow, blood–brain barrier integrity, and neuroinflammation regulation.

Building upon this foundation, the present study aims to evaluate the NO-mediated neuroprotective effects of nebivolol during the acute phase of MI. We hypothesize that the unique pharmacological profile of nebivolol will reduce acute brain changes following MI through NO-dependent mechanisms. This effect may offer a novel therapeutic strategy for preventing the neurological complications that contribute to long-term morbidity and mortality in MI patients.

## 2. Materials and Methods

### 2.1. Animals

Male Sprague-Dawley rats (250–300 g; 12 weeks old) were used in this study. Animals were housed individually and maintained under standard laboratory conditions (20–22 °C, 12/12 h light–dark cycle, free access to food and water). Sample sizes were determined through prior statistical analyses to minimize animal use in accordance with the 3Rs principles. Animals were maintained in pathogen-free conditions and handled following the ARRIVE guideline [16]. All experimental procedures were approved by the local ‘Institutional Animal Ethics Committee’.

### 2.2. Experimental Groups

Animals were randomly divided into four groups: sham-operated control (sham-control); MI-induced control (MI-control); MI-induced and oral nebivolol-treated group (MI-neb1); and MI-induced and immediate intravenous load followed by oral nebivolol-treated group (MI-neb2). The sham-control group did not receive any treatment while MI-induced animals received solvent or nebivolol treatment, respectively.

### 2.3. Study Drug and Dose Regimens

The nebivolol loading dose was determined from our previous study [14], based on the literature [17,18], in which three doses (0.1, 0.5, and 1.0 mg/kg) were administered intravenously within 10 min following LAD ligation. Blood pressure and heart rate were monitored for 60 min and compared to sham-control animals. Based on these findings, 0.1 mg/kg was chosen as the loading dose to prevent impairment of coronary perfusion. The continuation dose was selected as 2 mg/kg. [14].

For the loading dose, nebivolol was dissolved in distilled water/polyethylene glycol (80:20, *v*/*v*) at a concentration of 0.2 mg/mL. The weight-adjusted volume was infused via the femoral artery at 0.2 mL/min. The continuation dose, prepared as a suspension in tap water, was administered once daily by oral gavage [14].

### 2.4. Induction of MI

MI was induced by ligating the left anterior descending coronary artery (LAD) as described previously [14]. Briefly, animals were anesthetized with ketamine (100 mg/kg) and xylazine (4 mg/kg); an anterior thoracotomy was performed under artificial ventilation (TOPO ventilator, Kent Scientific, Torrington, CT, USA), the heart was rapidly exteriorized, and LAD was ligated approximately 2 mm from its origin using a 6-0 suture. The lungs were fully inflated with positive pressure, and the chest was closed in layers. Following extubation and restoration of spontaneous breathing, mechanical ventilation was discontinued. Animals remained normothermic on a heating pad, and the tracheal incision was closed. Buprenorphine (0.1 mg/kg) was administered intraperitoneally for postoperative analgesia. Animals were maintained on a heat blanket set to 30 °C for 30 min and continuously monitored for any life-threatening conditions (excessive dyspnea or hemorrhage). The sham-control group underwent the same procedure without LAD ligation. All surgical procedures were performed under sterile conditions.

### 2.5. Evaluation of Cardiac Function

Cardiac functions were evaluated by transthoracic echocardiography as described previously [14]. Two-dimensional M-mode and pulse-wave Doppler echocardiographies were performed using an echocardiographic system equipped with a 10 MHz sector probe (General Electric, System Five, Horten, Norway). All measurements and calculations were performed in accordance with the standards set by the American Society of Echocardiography [19].

### 2.6. Hemodynamic Measurement

Hemodynamic measurements were performed following the method outlined in our previous work. In brief, a heparinized water-filled polyethylene-tubing catheter (PE-50), connected to the pressure transducer (MLT 0699, PowerLab, ADI Instruments, Oxford, UK), was inserted into the right carotid artery and advanced to the ascending aorta. After recording the aortic pressure on a physiological recorder (10T Hardware System, PowerLab, ADI Instruments, Oxford, UK), the catheter was advanced to LV and ventricular pressures were recorded.

### 2.7. Biologic Assessment

*Sample preparation:* Brain tissues were harvested from rats across different experimental groups. The cerebral cortex (CC), hippocampus (HC), and paraventricular nucleus (PVN) of the hypothalamus were carefully dissected from the brains on ice and immediately snap-frozen in liquid nitrogen for subsequent analysis.

*Cytokine measurement:* Proinflammatory cytokines (IL-1β, IL-6, and TNF-α) in the brain tissue were measured using the enzyme-linked immunosorbent assay (ELISA). The cerebral cortex (CC), hippocampus (HC), and PVN puncher of the brain were homogenized in ice-cold lysis buffer. The homogenates were centrifuged (15 min at 50,000 rpm), and the supernatant was collected. Protein content was assayed using the bicinchoninic (BCA) procedure. The cytokine content of the samples was assayed using commercial kits (RAB0480 Sigma Aldrich, St. Louis, MO, USA; ERA32RB, Thermo-Scientific, Waltham, MA, USA; ab100768, Abcam, Cambridge, UK) in accordance with the manufacturer’s instructions. The cytokine content was expressed at picograms of cytokine per milligram.

*Determination of oxidative damage and antioxidant capacity:* Oxidative damage and antioxidant capacity were assessed by measuring malondialdehyde (MDA) levels and superoxide dismutase (SOD) activity, respectively. Brain sections were weighed, washed in 0.9% NaCl, and homogenized in ice-cold 0.15 M 10% KCl (*w*/*v*). Homogenates of 20% were obtained and sonicated two times at 30 sec intervals at 4 °C. Homogenates were centrifuged at >10,000× *g* for 15 min. at 4 °C. Tissue Cu-Zn superoxide dismutase (Cu-Zn SOD) activity was determined by the method of Sun et al. by inhibition of nitro blue tetrazolium reduction, with xanthine/xanthine oxidase used as a superoxide generator. One unit of SOD was defined as the amount of protein that inhibits the rate of nitro blue tetrazolium reduction by 50% [20]. MDA levels were measured in homogenates by the thiobarbituric acid reactivity assay as previously described [21]. The total protein concentration was measured by the method of Lowry et al. [22].

### 2.8. Histopathological Assessment

Following hemodynamic measurements, the catheter was withdrawn to the aorta, and cardiac arrest was induced in diastole with iv KCl injection (3cc, 10% solution). The thorax was opened quickly, and the right atrium was incised to allow blood drainage. The heart and brain were perfused–fixed with 10% phosphate-buffered formalin at a constant pressure of 7.5 cm H_2_O for 1 h. After fixation, tissues were excised quickly, weighed, dehydrated, and embedded in paraffin.

*Tissue damage:* Hematoxylin and eosin (HE) staining was used to evaluate tissue damage in both cardiac and brain tissues. Paraffin-embedded tissues were sectioned transversely at 5 μm thickness. The HE staining protocol included deparaffinization in xylene, followed by rehydration through a graded ethanol series (100%, 95%, 80%, and 70%) and double-distilled water. Sections were then immersed in hematoxylin for 5 min, rinsed twice with double-distilled water, differentiated in 0.1% acid alcohol solution, and rinsed in tap water for 15 min. Subsequently, sections were immersed in eosin for 1 min, rinsed in tap water, dehydrated through graded ethanol (95% and 100%) and xylene, and mounted using resinous mounting medium. Histological images were captured using a light microscope (Nikon Eclipse E100, Tokyo, Japan) and evaluated by two independent blinded observers.

*Infarct Size:* The 10 μm-thin sections were serially cut from the apex to the base at 1 mm intervals, deparaffinized, and stained with HE and Masson’s trichrome (MS). Slices were scanned using a digital slide scanner (Minato, Tokyo, Japan) at 20× magnification to obtain an image of the complete heart section. The infarct was outlined, and the total area of the infarct section was digitally determined by the software (NIS-Elements L imaging software, ToupTek. XCamview, Version: V1.6_20210122). Infarct size (fraction of the infarcted left ventricle) was calculated as the average of all slices [23]. Infarct size quantification was performed by two independent blinded observers.

### 2.9. İmmunohistochemistry

**Figure 1:**
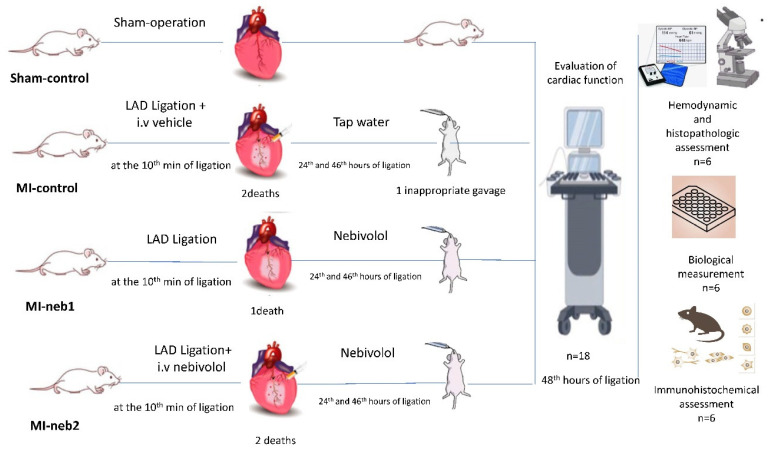
Experimental procedure.

Following deparaffinization and rehydration, brain tissue sections were subject to heat-mediated antigen retrieval using EDTA antigen retrieval buffer (pH 9.0) (Bond Epitope Retrieval 2 RTU, Leica, London, UK) for 30 min in accordance with the Leica ER2 standard protocol. The sections were then incubated for 45 min at room temperature with the following antibodies: rabbit-anti-eNOS antibody (1:300, ZRB1252, Sigma, St. Louis, MO, USA) and rabbit-anti-iNOS antibody (1:300, ZRB1449, Sigma, St. Louis, MO, USA). Immunoreactivity was visualized using the Leica Bond III device (Leica, London, UK) with the DAB Kit (DS9800, Bond Polymer Refine Detection Kit, Leica, London, UK). For negative controls, adjacent sections were processed following the same steps, except for the primary antibodies. All sections were examined under a light microscope (Olympus BX51, Tokyo, Japan). Immunolabelling intensity was assessed using a semi-quantitative scale: 1 = weak, 2 = moderate, and 3 = strong. Two independent blinded observers evaluated the slides, and inter-rater consistency was ensured through repeated calibration. A schematic overview of the experimental procedure is given in Figure 1.

### 2.10. Statistical Analysis

All data are presented as mean ± SD. Differences among groups were assessed using one-way analysis of variance (ANOVA) followed by Scheffe’s post hoc test. A value of *p* < 0.05 in a two-tailed distribution was considered as statistically significant.

## 3. Results

A total of 78 animals were included in the study. There were no deaths in the sham-control group. In MI-induced groups, mortality rates were 12%, 9%, and 10% for MI-control, MI-neb1, and MI-neb2 groups, respectively. Deaths occurred within the first 24 h following MI induction.

**Figure 2:**
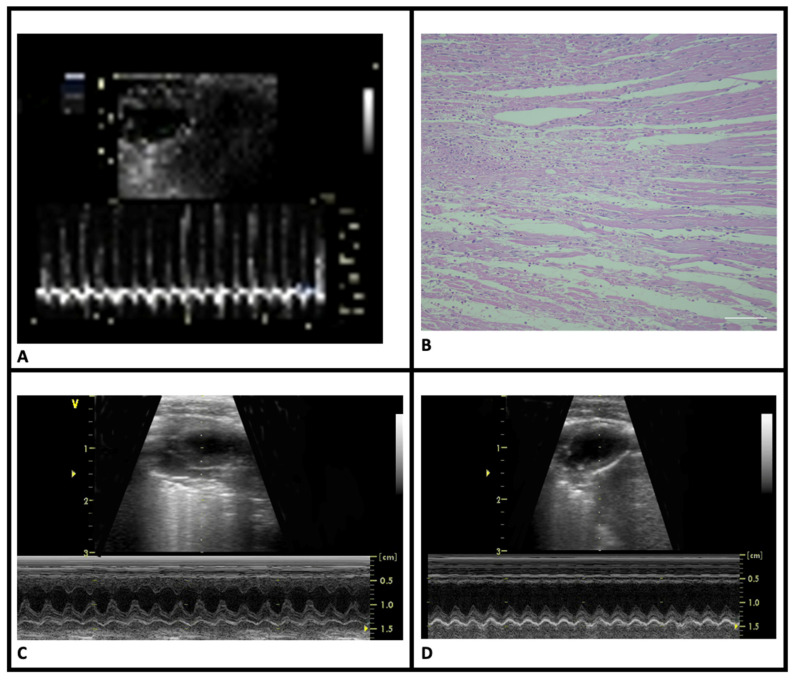
Clinical and histopathological features of myocardial infarction. (**A**) ST-segment elevation on electrocardiogram tracings of MI-induced groups. (**B**) Coagulative necrosis in the HE-stained sections of MI-induced animals. (**C**) Normal wall motion of the sham-control group in M-mode echocardiography images. (**D**) Significant akinesia in the anterior wall of the MI-control group in M-mode echocardiography images (HE, scale bar = 20 μm).

*Clinical verification of MI*: Following ligation of the LAD, the myocardium distal to the ligation site turned purple or pale, and the heartbeat weakened with visible congestion of the left auricle. MI was confirmed via an ECG, which showed ST elevation (Figure 2A).

*Histopathological verification of MI*: HE staining revealed pathological changes in LV consistent with acute MI. Compared to the sham-control group, the MI groups displayed signs of acute infarction, including eosinophilia, karyolysis, leukocyte infiltration, and disorganization of muscle fibers. Infarct regions were primarily located in the anterolateral LV, extending from the sub-endocardial to endocardial area (Figure 2B). There were no statistically significant differences in infarct sizes between the MI groups (Table 1).

*Cardiac functions:* Echocardiographic assessments of LV geometry and function are shown in Table 1. Compared to the sham-control group, MI-control rats exhibited significant LV structural changes, including thickening of the remote non-infarct myocardial wall (Figure 2C,D). Functional abnormalities characterized by decreased ejection fraction (EF) consistent with structural changes were observed in this group. Nebivolol treatment preserved both LV structure and function in the MI-neb1 and MI-neb2 groups.

*Hemodynamic parameters:* Compared to the sham-control, the MI-control group of animals exhibited decreased mean arterial pressure (MAP) and increased ∆±dp/dt and left ventricular end-diastolic pressure (LVEDP). While nebivolol treatment slightly decreased MAP, it effectively prevented the MI-associated increase in LVEDP and decrease in ∆±dp/dt in the MI-nebivolol groups (Table 2).

*Tissue oxidative damage and antioxidant capacity:* Brain SOD and MDA levels of the four groups are shown in Table 3. In the MI-control group, increased MDA levels in the CC, HC, and PVN were accompanied by decreased SOD levels (*p* < 0.05 for all comparisons). Nebivolol treatment mitigated these changes, particularly in the MI-neb2 group, where intravenous administration significantly restored both MDA and SOD levels (*p* < 0.05, compared to MI-control group).

**Figure 3:**
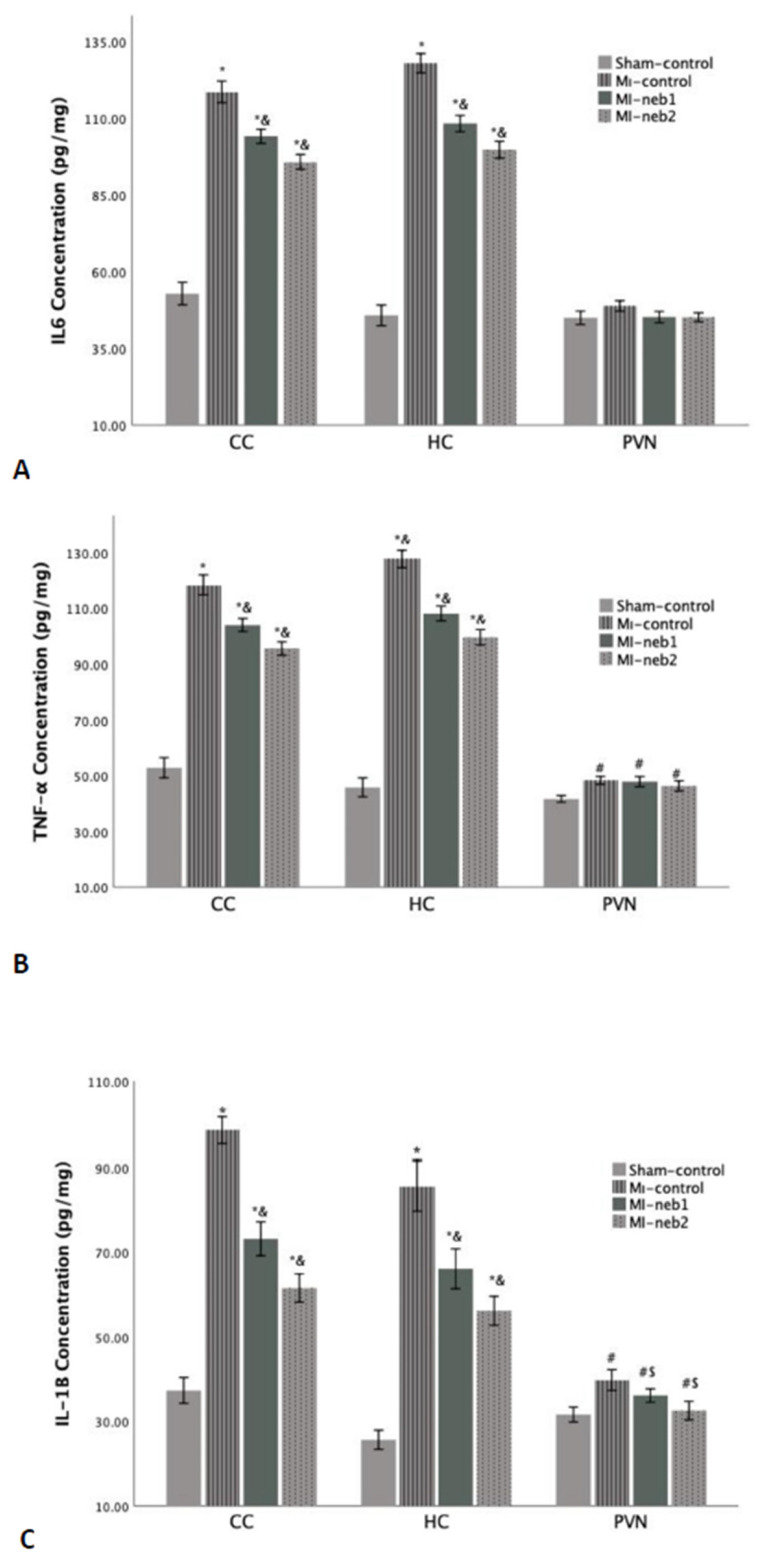
Proinflammatory cytokine levels in the brain sections. L-1β concentrations in all three brain regions of the MI-induced groups are high (**A**). However, both IL-6 (**B**) and TNF-α levels (**C**) are significantly high in the CC and HC regions of MI-induced groups. * *p* < 0.0001 compared to Sham-control; & *p* > 0.01 compared to MI-control; # *p* < 0.05 compared to Sham-control; S *p* < 0.05 compared to MI-control; CC: Cerebral cortex; HC: Hippocampus; PVN: Paraventricular nucleus).

*Inflammatory response:* Proinflammatory cytokine levels in the brain are shown in Figure 3. In MI-control rats, IL-1β, IL-6, and TNF-α levels were markedly elevated in the HC and CC (*p* < 0.001). In nebivolol-treated groups, proinflammatory cytokine levels in these two brain regions were significantly lower than in the MI-control group (*p* < 0.001 for all comparisons).

**Figure 4:**
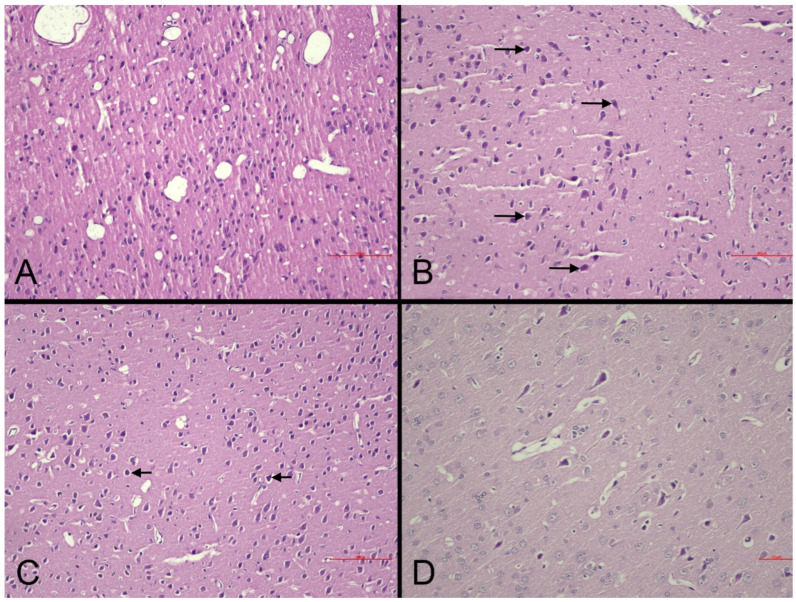
Representative photomicrographs of the cerebral cortex in experimental groups (HE). (**A**): Sham-control group. Normal cortical cytoarchitecture and neuronal morphology are preserved (scale bar = 90 µm). (**B**): MI-control group. Marked neuronal degeneration/necrosis (arrows), perineuronal vacuolation, and partial disorganization of laminar structure (scale bar = 90 µm). (**C**): MI-neb1 group. Mild neuronal degeneration (arrows) with occasional necrotic neurons; overall laminar organization preserved (scale bar = 90 µm). (**D**): MI-neb2 group. Nearly normal cortical appearance with well-preserved neuronal morphology and minimal degenerative changes (scale bar = 50 µm).

*Histopathological changes in the brain:* Microscopic examination of brain sections demonstrated necrotic alterations in the CC and HC (Figure 4). Standard HE staining did not reveal distinctive histopathological changes in the PVN. Animals in the MI-control group displayed more severe necrosis (+2 score) compared to sham-controls (+1 score) within the CC (Figure 4A,B), confirming that necrotic damage is directly associated with pathophysiological mechanisms following MI. Necrosis severity in nebivolol-treated groups remained equivalent to the MI-control group (+2 score across all groups) in the CC (Table 4). The similarity in necrosis levels between nebivolol-treated and MI-control groups indicates that nebivolol does not provide substantial protection against cortical necrosis during the acute post-MI phase (Figure 4C).

Within the HC, MI-control animals demonstrated moderate necrosis (+2 score) across all subregions, while the MI-neb1 group showed moderate necrosis (+2 score) specifically in the CA1 and CA3 subfields. Neither the sham-control nor MI-neb2 groups exhibited necrotic changes in the HC (Table 4).

MI-control animals were characterized by increased reactive astrocytosis (+2 score). In contrast, both the sham-control and MI-neb2 groups exhibited no astrocytosis (0 score). The MI-neb1 group exhibited a partial effect (+1 score) (Table 4). The presence of astrocytosis in the MI-induced groups indicates that a neuroinflammatory response occurred in the brain following MI. The absence of astrocytosis in the sham-control and MI-neb2 groups suggests that parenteral nebivolol may have suppressed this neuroinflammatory response.

Increased glial cellularity, consistent with gliosis, was identified in the MI-control and MI-neb1 groups, whereas no gliotic changes were observed in the sham-control animals (Figure 4). The significant gliosis observed in the MI-control group suggests that brain tissue increases glial proliferation in response to injury. Gliosis in the MI-neb2 group was similar to that in the sham-control group, suggesting that parenteral nebivolol may have a suppressive effect on the glial response.

Demyelination was observed in both MI-control and MI-neb1 groups (+1 score for both groups) but was absent in sham-control and MI-neb2 groups (Table 4). The presence of demyelination in the MI-control and MI-neb1 groups, but its absence in the MI-neb2 and sham-control groups, suggests that axonal damage after MI persisted, especially in the oral nebivolol group, although parenteral nebivolol may have suppressed this process.

**Figure 5:**
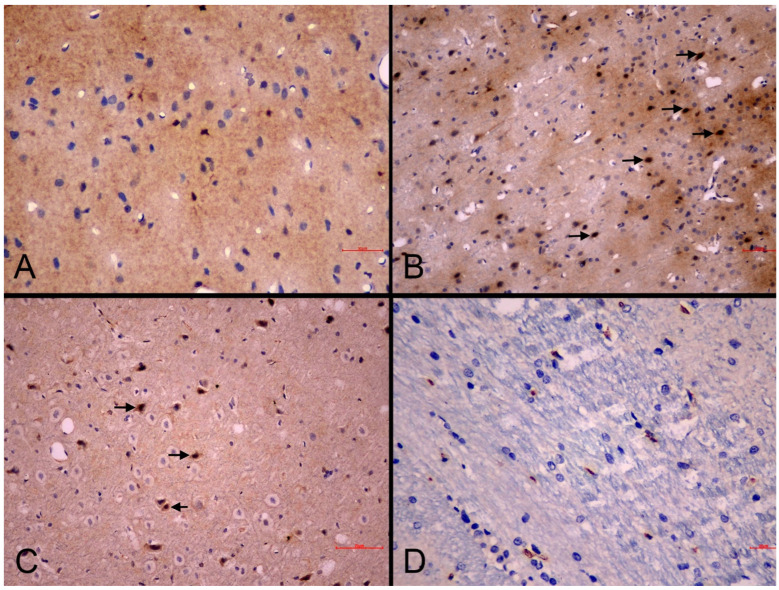
Representative photomicrographs of iNOS immunoreactivity in the cerebral cortex of experimental groups. (**A**): Sham-control group. Mild cytoplasmic iNOS immunoreactivity in scattered neurons (IHC, scale bar = 30 µm). (**B**): MI-control group. Strong cytoplasmic iNOS immunoreactivity in neurons and glial cells (arrows) (IHC, scale bar = 50 µm). (**C**): MI-neb1 group. Moderate cytoplasmic iNOS immunoreactivity in neuronal cell bodies (arrows) (IHC, scale bar = 70 µm). (**D**): MI-neb2 group. Weak iNOS immunoreactivity (IHC, scale bar = 20 µm).

*NOS immunoreactivity in the brain:* Immunohistochemical analysis of nitric oxide synthase (NOS) isoforms revealed distinct expression patterns. iNOS immunoreactivity was assessed via cytoplasmic staining in astrocytes, microglia, and endothelial cells (Figure 5), while eNOS was evaluated within vascular endothelial cells.

**Figure 6:**
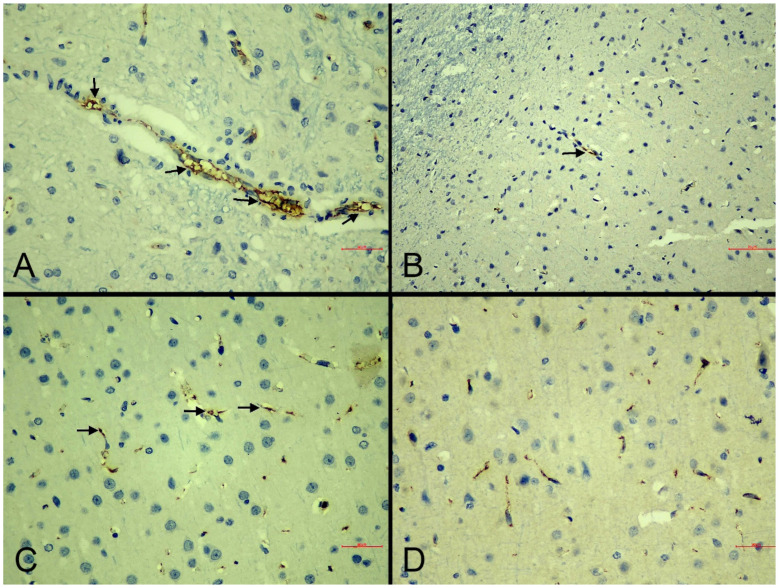
Representative photomicrographs of eNOS immunoreactivity in the cerebral cortex of experimental groups. (**A**): Sham-control group. Strong, continuous eNOS immunoreactivity in vascular endothelial cells (arrows) (IHC, scale bar = 30 µm). (**B**): MI-control group. Mild and discontinuous eNOS immunoreactivity (arrows) (IHC, scale bar = 70 µm). (**C**): MI-neb1 group. Moderate endothelial eNOS immunoreactivity (arrows) indicating partial restoration (IHC, scale bar = 30 µm). (**D**): MI-neb2 group. Strong eNOS immunoreactivity (IHC, scale bar = 30 µm).

eNOS immunolabeling intensities showed no statistically significant differences between experimental groups. Mild to moderate positive staining was detected even in some sham-control samples, indicating that eNOS maintained physiological expression levels across all treatment conditions (Figure 6).

The sham-control group demonstrated mild iNOS expression (extent and intensity score of +1), while the MI-control group exhibited enhanced immunolabeling intensity (extent score of +2, and intensity +3) compared to sham-controls, suggesting a potential association between glial cell activation and upregulated iNOS expression following MI (Table 5).

Treatment effects varied between the two intervention groups. The MI-neb1 group maintained elevated iNOS labeling intensity similar to MI-control animals (extend and intensity score of +2) while the MI-neb2 group showed attenuated iNOS expression (extend and intensity score of +1) (Figure 4A,B), indicating that intravenous nebivolol may suppress MI-induced iNOS upregulation (Table 5).

## 4. Discussion

This investigation presents new evidence establishing nebivolol’s neuroprotective capacity during acute MI through NO-mediated pathways. Our data reveal several key findings: (1) robust inflammatory responses emerge in both the CC and HC during the acute post-MI phase following LAD ligation, (2) nitrosative stress constitutes the predominant pathophysiological driver of neurodegeneration within the CC and HC, and (3) intravenous nebivolol administration markedly reduces neuroinflammation, oxidative/nitrosative stress, and structural cerebral alterations, whereas oral delivery demonstrates minimal protective benefit, indicating route-specific therapeutic efficacy.

These observations substantiate the concept that nebivolol’s distinctive pharmacological characteristics, integrating β1-adrenergic antagonism with NO-releasing activity, provide neuroprotective benefits that complement its cardioprotective effects during the acute post-MI phase.

Cardiovascular diseases represent the primary cause of death and illness worldwide [24]. MI constitutes the most serious manifestation of coronary artery disease, accounting for approximately 2.4 million fatalities annually in the United States, over 4 million deaths in Europe and northern Asia, and representing more than one-third of all deaths in developed nations [25]. Although extensive research has been conducted on cardiac pathophysiology, the consequences of cardiovascular disease on neurological function represent a critical yet insufficiently investigated domain.

The brain–heart axis is a bidirectional communication network involving autonomic, hormonal, and immune pathways, through which the brain regulates cardiac function and, conversely, cardiac events influence brain health [26]. Neurological conditions such as cognitive impairment, migraines, and epilepsy have been linked to coronary artery disease, heart failure, and congenital heart abnormalities [27]. Notably, MI is also frequently associated with brain injuries [12] and behavioral disorders, like anxiety, depression, and dementia [28,29].

MI triggers a complex cascade of neurological and cardiovascular interactions. This connection becomes particularly important after an MI, as neural regulatory mechanisms attempt to compensate for cardiac damage while the brain itself may be adversely affected by the cardiac event. These mechanisms include alterations in hemodynamics, the autonomic nervous system, baroreflex sensitivity, the renin–angiotensin–aldosterone system, sarcoplasmic reticulum calcium transient, the beta-adrenergic pathway, inflammation, and oxidative stress, which are observed in both human subjects and experimental models [30].

***Robust inflammatory responses emerge in both the CC and HC following MI:*** Extensive research demonstrates that MI triggers robust activation of inflammatory pathways [31]. This inflammatory response, which is essential for cardiac tissue remodeling and scar tissue development, extends beyond the heart to affect systemic circulation [32]. Evidence suggests that this systemic inflammatory response can rapidly induce neuroinflammation within minutes of MI onset [6], with neuroinflammatory changes potentially persisting for extended periods of approximately 6–8 weeks [33]. Neuroinflammation is mediated by the production of cytokines, chemokines, and reactive oxygen/nitrogen species, which are produced by microglia, astrocytes, peripherally derived immune cells, and endothelial cells [34,35].

Microglia function as dynamic surveillance cells with diverse roles in both physiological and pathological states [36]. In their resting state, these cells exhibit a compact soma with extensively branched, thin projections and minimal expression of cell surface markers [37]. Upon encountering tissue damage or inflammatory signals, microglia undergo dramatic morphological changes, increase in number, secrete proinflammatory mediators, and upregulate various immunomodulatory surface molecules [38]. Studies employing cardiac ischemia/reperfusion models have revealed that microglial activation undergoes a shift from initially neuroprotective responses to harmful inflammatory states [39]. Preclinical investigations have consistently documented elevated concentrations of proinflammatory cytokines, specifically tumor necrosis factor-α (TNF-α), interleukin-6 (IL-6), and interleukin-1 (IL-1), accompanied by microglial activation across multiple brain regions including CC and HP within 24 h following MI [40,41]. Following permanent left anterior descending (LAD) coronary artery ligation in rats, researchers observed substantial increases in activated microglia within the paraventricular nucleus (PVN) at 4–6 weeks post-MI. These findings suggest that microglial activation in the PVN follows a delayed onset rather than occurring immediately after MI, becoming apparent over time and contributing to the persistent elevation of cytokine levels characteristic of the post-MI period [42]. Nonetheless, some studies have also documented early microglial activation following MI [43].

In addition to microglia, activation of astrocytes in different regions of the brain such as the amygdala and PVN after an MI was shown [44]. Moreover, the decrease in MI-induced expression of proinflammatory cytokines (TNF-α and IL-6) was shown to occur by the inhibition of astrocytes in the PVN [45]. Similarly, early astrocytic activation was observed in the rat model of cardiac ischemia/reperfusion [39].

Neuroinflammation resulting from physical injury has been identified as a key contributor to behavioral changes, including anxiety, depression, and impaired cognitive function [46,47]. Studies have shown that microglia become activated in animal models of stress-related depression, indicating that microglial activity may be central to the emergence of depressive behaviors during the chronic period following MI [48]. Using a mouse model of cardiac ischemia–reperfusion injury, Evonuk and colleagues demonstrated a direct link between elevated microglial populations in the dentate gyrus (DG), CA1, and CA3 regions of the hippocampus (HC) at 72 h post-injury and both impaired neurogenesis and deficits in hippocampal-dependent learning and memory [49]. Supporting these observations, research using permanent left anterior descending (LAD) coronary artery ligation has shown increased microglial numbers and activation in the DG of the HC in mice, with these alterations associated with declining cognitive performance [41].

Our findings align with previous research, demonstrating a pronounced neuroinflammatory response following MI characterized by significantly elevated proinflammatory cytokine concentrations (IL-1β, IL-6, and TNF-α) accompanied by distinct structural pathological changes, including necrosis, astrocytosis, and gliosis within the CC and HC (especially in the DG, CA1, and CA3 subregions). These observations provide evidence for direct cerebral involvement subsequent to cardiac injury and offer mechanistic insight into the well-documented increased incidence of stroke, anxiety, depression, and cognitive dysfunction following MI. Our results are further supported by recent clinical neuroimaging studies, which demonstrate that acute coronary syndrome-induced neuroinflammation produces measurable neurological alterations in brain regions essential for memory processing and neuroendocrine regulation [50].

***Nitrosative stress constitutes the predominant pathophysiological driver of neurodegeneration within the CC and HC:*** During aerobic respiration, mitochondria produce free radicals as natural byproducts when they reduce molecular oxygen [51]. Although these radicals play essential physiological roles, excessive oxidative and nitrosative stress becomes problematic when it exceeds the body’s protective capacity. Such damaging conditions emerge when antioxidant defense systems fail to sufficiently counteract reactive oxygen species (ROS) or reactive nitrogen species (RNS) [52]. The nervous system faces heightened vulnerability to oxidative damage because neurons require substantial amounts of oxygen for metabolism yet possess comparatively weak antioxidant defenses. Elevated ROS and RNS concentrations initiate destructive cascades that oxidize proteins and lipids, damage DNA, and ultimately cause neuronal dysfunction and cell death [53].

Nitric oxide (NO) is a gaseous molecule generated through the enzymatic conversion of L-arginine to L-citrulline by nitric oxide synthase (NOS) enzymes. Three distinct NOS isoforms produce NO: two constitutive forms (nNOS and eNOS) and one inducible form (iNOS). The constitutive isoforms generate steady, rhythmic pulses of NO under normal physiological conditions, whereas iNOS is activated during inflammatory responses to produce substantially higher concentrations of NO [54].

Cytokine (IL-1α, IL-1β, TNF-α, IFN-γ)-induced NO release via iNOS in glial cells is known [55]. Elevated NO levels from upregulated iNOS cause nitrosylation of cysteine residues in arginase, enhancing arginase activity and depleting L-arginine stores. This L-arginine depletion leads to eNOS uncoupling, transforming eNOS into a radical-producing enzyme that generates superoxide anions (•O2^−^). The subsequent reaction between NO and •O2^−^ forms peroxynitrite (ONOO^−^), a highly reactive species with superior membrane penetration compared to •O2^−^ [56]. Once inside cells, ONOO^−^ initiates widespread oxidative damage through protein tyrosine nitration, lipid peroxidation, mitochondrial dysfunction, and DNA strand breaks [57], ultimately causing neuronal death and tissue damage [58]. This neuronal death creates a positive feedback loop by further stimulating NOS expression and NO production, perpetuating the destructive cycle.

In our study, the substantial necrosis observed both in the CC and HC of MI-control rats demonstrates that neuronal death directly correlates with the pathophysiological cascade initiated by MI. The elevation of iNOS expression alongside normal eNOS levels, combined with reduced SOD activity in this group, suggests that nitrosative damage represents the main pathophysiological mechanism underlying neurodegeneration in these brain regions. These findings align with previous reports by Yoshida et al. regarding cerebrovascular injury [59]. Recent studies have additionally demonstrated MI-induced oxidative damage in the hippocampus [60] and established MI-associated reactive oxygen species as contributors to cognitive impairment [61].

Beyond their direct effects on glial cells, ROS/RNS directly compromise myelin integrity by attacking both lipid and protein components of the myelin sheath [62,63], while concurrently facilitating macrophage-mediated degradation. Exposure to ROS triggers lipid peroxidation within myelin and causes structural disruption of myelin lamellae at the intraperiod line. Furthermore, peroxynitrite modifies low-density lipoprotein into a configuration that activates macrophage scavenger receptors [64], hereby amplifying the inflammatory response. The vulnerability of white matter to hypoperfusion is well-documented in ischemic stroke, where profound white matter injury routinely occurs [65]. Evidence demonstrates that cerebral oxygen saturation declines as early as 4 h following LAD ligation in mice models [66]. Our observations of extensive demyelination, increased MDA concentrations, and diminished MAP align with the existing literature indicating that white matter, primarily consisting of myelinated axons, displays exceptional sensitivity to reduced perfusion. This inherent susceptibility produces disproportionately severe white matter injury relative to gray matter, consequently resulting in more pronounced neurological deficits.

***Nebivolol is a promising candidate for dual-action therapy after MI:*** Although our study focused on nebivolol, neuroprotection after MI is an emerging therapeutic target. Several pathways have been proposed, including sympathetic and parasympathetic nervous systems, the renin–angiotensin–aldosterone axis, hypothalamic–pituitary–adrenal axis, microRNA, and cytokines [12]. Our findings extend current knowledge by showing that an approved beta-blocker not only preserves cardiac function but also provides tangible neuroprotection, making it a promising candidate for dual-action therapy. This is especially important given that cardiovascular disorders are the second leading cause of death after stroke, underscoring the need for treatments that address both heart and brain complications at the same time.

Cardiac outcomes observed in our study are consistent with the existing literature on the cardioprotective effects of nebivolol. As anticipated, both oral and parenteral nebivolol administration preserved left ventricular geometry and function, with improvements in echocardiographic and hemodynamic parameters and, more importantly, without affecting infarct size. This suggests that its benefits are not attributed to reduced ischemic burden but likely result from modulation of downstream pathophysiological processes.

The anti-inflammatory effects of nebivolol we observed are consistent with the broader literature on β-blocker therapy. Recent studies have shown that sympathetic nerve blockade, notably via the administration of beta-blockers, reduces circulating levels of several proinflammatory cytokines, such as TNF-α, IL-1β, and IL-6, after injury [26,67]. Our findings extend these observations by demonstrating that the anti-inflammatory effects of nebivolol extend to brain tissue, not just systemic circulation. The observed mild iNOS expression, together with physiological eNOS levels in the MI-neb2 group, suggests that nebivolol may modulate the balance between beneficial and detrimental NO production. This selective modulation of NOS isoforms may contribute to an overall neuroprotective effect by maintaining physiological NO levels while preventing excessive inflammatory NO production. Demonstration of antioxidant and neuroprotective properties of nebivolol in different brain pathologies supports our findings [68,69,70].

The differential efficacy between intravenous and oral administration we observed is particularly noteworthy. While oral nebivolol (MI-neb 1) showed limited neuroprotective effects, intravenous administration (MI-neb2) significantly reduced neuroinflammatory markers, gliosis, and oxidative/nitrosative stress. This difference may be attributed to rapid modulation of proinflammatory signaling, which plays a critical role in cerebral injury following MI.

## 5. Conclusions

This study demonstrates that nebivolol, particularly when administered intravenously, provides significant neuroprotective effects during the acute phase of MI. The drug effectively attenuates neuroinflammation, reduces ROS/RNS, and prevents structural brain changes through NO-dependent mechanisms.

Future research should focus on clinical translation of these findings, including dose optimization, timing of administration, and long-term neuroprotective outcomes. Given the growing recognition of brain complications in cardiovascular disease, interventions like nebivolol may represent a promising dual-action strategy to improve both cardiac and neurological recovery following MI.

## Figures and Tables

**Table 1:** Left ventricular structure and functions.

Parameter	Sham-Control (*n* = 18)	MI-Control (*n* = 18)	MI-neb1 (*n* = 18)	MI-neb2 (*n* = 18)
LVEDd (cm)	0.63 ± 0.09	0.65 ± 0.07	0.60 ± 0.06	0.62 ± 0.04
LVEDv (mL)	0.69 ± 0.04	0.79 ± 0.06 ^#^	0.73 ± 0.12	0.72 ± 0.09
EF (%)	70.17 ± 6.20	58.17 ± 7.90 ^#^	66.17 ± 3.98 ^#,&^	64.28 ± 2.18 ^#,&^
CO (mL/min)	756 ± 269	724 ± 210 ^#^	762 ± 214 ^&^	760 ± 236 ^&^

(*p* < 0.05 ^#^ compared to sham-control group and ^&^ compared to MI-control group). LVEDd: Left ventricular end-diastolic dimension; LVEDv: left ventricular end-diastolic volume; EF: left ventricular ejection fraction; CO: cardiac output.

**Table 2:** Hemodynamic parameters and infarct sizes.

	Sham-Control (*n* = 6)	MI-Control (*n* = 6)	MI-neb1 (*n* = 6)	MI-neb2 (*n* = 6)
Infarct size (%)	-	49.2 ± 4.34	46.9 ± 5.02	47.2 ± 3.12
MAP (mmHg)	119.1 ± 7.4	93.5 ± 8.8 ^#^	106.3 ± 10.0 ^#^	102.2 ± 6.5 ^#^
LVEDP (mmHg)	2.0 ± 0.23	28.3 ± 4.1 ^#^	12.5 ± 4.0 ^#,&^	13.6 ± 2.8 ^#,&^
Δ+dp/dt (mmHg/min)	6716 ± 574	4078 ± 411 ^#^	4613 ± 291 ^#,&^	4572 ± 256 ^#,&^
Δ−dp/dt (mmHg/min)	5308 ± 595	2691 ± 346 ^#^	3354 ± 339 ^#,&^	3028 ± 215 ^#,&^

(*p* < 0.05 ^#^ compared to sham-control group and ^&^ compared to MI-control group). MAP: Mean arterial pressure; LVEDP: Left ventricle end-diastolic pressure; Δ+dp/dt: Maximum rise in left ventricle pressure; Δ−dp/dt: Maximum fall in left ventricle pressure.

**Table 3:** Brain oxidative stress and antioxidant capacity.

Group (*n* = 6)	SOD Levels (U/mg Protein)			MDA Levels (nmol/mg Tissue)		
	CC	HC	PVN	CC	HC	PVN
Sham-control	4.53 ± 0.19	7.34 ± 0.28	5.36 ± 0.32	4.52 ± 0.53	3.68 ± 0.41	2.14 ± 0.23
MI-control	3.21 ± 0.51 ^#^	5.74 ± 0.31 ^#^	4.82 ± 0.45 ^#^	7.63 ± 0.34 ^#^	6.14 ± 0.23 ^#^	2.36 ± 0.42 ^#^
MI-neb1	4.18 ± 0.28 ^#,&^	6.97 ± 0.51 ^#,&^	4.98 ± 0.38 ^#,&^	4.21 ± 0.45 ^&^	3.33 ± 0.62 ^&^	1.98 ± 0.24 ^&^
MI-neb2	4.37 ± 0.37 ^&^	7.19 ± 0.34 ^&^	5.06 ± 0.23 ^&^	4.31 ± 0.26 ^&^	3.46 ± 0.43 ^&^	2.06 ± 0.14 ^&^

*p* < 0.05 ^#^ compared to sham-control group and ^&^ compared to MI-control group). CC: Cerebral cortex; HC: Hippocampus; PVN: Paraventricular nucleus.

**Table 4:** Histopathological characteristics.

Group (*n* = 6)	Necrosis			Astro Cytosis	Gliosis	Demyelination
	CC	HC				
	Score	Subfield	Score	Score	Score	Score
Sham-control	+1	-	-	-	-	-
MI-control	+2	DG, CA1, CA2, CA3	+2	+2	+2	+2
MI-Neb1	+2	CA1, CA3	+2	+2	+2	+2
MI-neb2	+2	-	-	-	-	-

CC: Cerebral cortex; HC: Hippocampus; CA: Corni ammonis; DG: Dentate gyrus.

**Table 5:** iNOS Immunolabeling scores.

Labeling Characteristics	Group (*n* = 6)	Score	
		Extent	Intensity
Cytoplasmic staining in astrocytes, microglia, and endothelial cells	Sham-control	+1	+1
	MI-control	+2	+3
	MI-neb1	+2	+2
	MI-neb2	+1	+1

## Data Availability

All data generated or analyzed during this study are included in this published article.
